# Mechanism of the trivalent lanthanides’ persistent luminescence in wide bandgap materials

**DOI:** 10.1038/s41377-022-00736-5

**Published:** 2022-03-08

**Authors:** Leipeng Li, Tianyi Li, Yue Hu, Chongyang Cai, Yunqian Li, Xuefeng Zhang, Baolai Liang, Yanmin Yang, Jianrong Qiu

**Affiliations:** 1 Hebei Key Laboratory of Optic-electronic Information and Materials, College of Physics Science & Technology, Hebei University, Baoding, 071002 China; 2 California NanoSystems Institute, University of California, Los Angeles, CA 90095 USA; 3 State Key Laboratory of Modern Optical Instrumentation, College of Optical Science and Engineering, Zhejiang University, Hangzhou, 310058 China

**Keywords:** Optics and photonics, Optical materials and structures

## Abstract

The trivalent lanthanides have been broadly utilized as emitting centers in persistent luminescence (PersL) materials due to their wide emitting spectral range, which thus attract considerable attention over decades. However, the origin of the trivalent lanthanides’ PersL is still an open question, hindering the development of excellent PersL phosphors and their broad applications. Here, the PersL of 12 kinds of the trivalent lanthanides with the exception of La^3+^, Lu^3+^, and Pm^3+^ is reported, and a mechanism of the PersL of the trivalent lanthanides in wide bandgap hosts is proposed. According to the mechanism, the excitons in wide bandgap materials transfer their recombination energy to the trivalent lanthanides that bind the excitons, followed by the generation of PersL. During the PersL process, the trivalent lanthanides as isoelectronic traps bind excitons, and the binding ability is not only related to the inherent arrangement of the 4f electrons of the trivalent lanthanides, but also to the extrinsic ligand field including anion coordination and cation substitution. Our work is believed to be a guidance for designing high-performance PersL phosphors.

## Abstract (web summary)

Long persistent luminescence of Gd^3+^ is reported and a possible mechanism of the trivalent lanthanides’ persistent luminescence in wide bandgap hosts is proposed.

## Graphical abstract

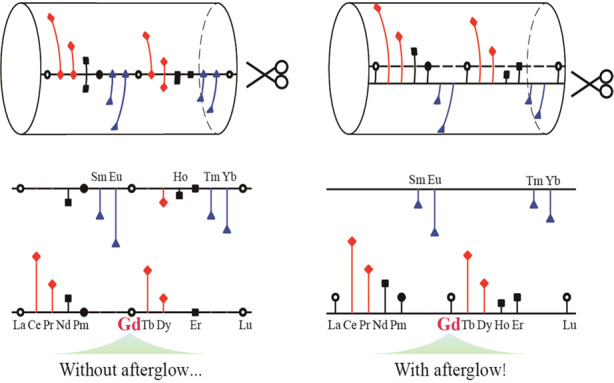

## Introduction

Persistent luminescence (PersL) phosphors can continue to emit light for seconds and even longer after the stoppage of excitation. Due to this attractive feature, PersL phosphors have a wide range of applications in the fields of display, anti-counterfeiting, information storage, biological labeling, etc.^1–8^. In general, luminescent center plays a key role in PersL phosphors. The commonly used luminescent centers that can generate PersL include lanthanide series, transition metal elements, and other ions such as Bi^3+^^9–15^. The latter two kinds of luminescent centers are generally limited to their relatively narrow emitting spectral bands, mainly in the visible spectral range. In contrast, the trivalent lanthanides are especially attractive as their 4f electrons are shielded by the outer 5s and 5p electrons and are thus less affected by the surrounding crystalline field, making the 4f ↔ 4f transitions feature narrow-band, high color purity and wide spectral range from the UV to NIR^16^. In spite of these attractive points, the previous literatures focus mainly on visible PersL of the trivalent lanthanides. To achieve PersL in the UV or even deep UV and NIR range is still a challenge, although there has been some related work^17–20^. Moreover, there are other problems such as how to tailor the intensity of PersL, how to obtain desirable excitation and emission bands, and so forth. Fundamentally, all these questions can be ascribed to the absence of a deep understanding on PersL or a more reasonable PersL model. Since the green phosphor of SrAl_2_O_4_:Eu^2+^,Dy^3+^ has been shown to own excellent PersL^21^, several underlying mechanisms have been put forward to explain PersL, including the hole trapping-detrapping model, the electron trapping-detrapping model, and the quantum tunneling model^1,2,22–25^. Although these models could explain some observed phenomena, there are flaws for these models and some key points still remain unclear. It is difficult to put forward a universal mechanism to reasonably explain all experimental results, and here we mainly focus on the PersL of the trivalent lanthanides in wide bandgap hosts due to its extensive application value^20^.

Here we show the trivalent lanthanides with the exception of La^3+^, Lu^3+^, and Pm^3+^ could generate PersL ranging from 200 to 1700 nm in the selected wide bandgap hosts NaYF_4_, Cs_2_NaYF_6_, *X*PO_4_ (*X* = Y, Sc, Lu, and La), and YBO_3_. Depending on the abundant experiments and analysis, a mechanism of the trivalent lanthanides’ PersL in wide bandgap hosts is proposed. It is found that the energy transfer from the excitons formed in wide bandgap materials to the trivalent lanthanides plays a key role for PersL. In addition, the trivalent lanthanides as isoelectronic traps bind excitons, and the binding ability not only depends on the inherent arrangement of 4f electrons of the trivalent lanthanides, but also on the extrinsic ligand field including anion coordination and cation substitution.

## Results

**Fig. 1:**
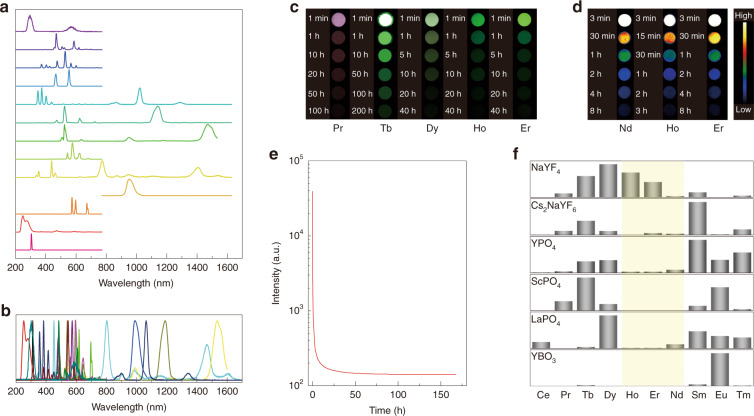
PersL of samples. **a** Normalized PersL spectra of NaYF_4_:Ce^3+^, NaYF_4_:Pr^3+^, NaYF_4_:Tb^3+^, NaYF_4_:Dy^3+^, NaYF_4_:Nd^3+^, NaYF_4_:Ho^3+^, NaYF_4_:Er^3+^, NaYF_4_:Sm^3+^, NaYF_4_:Tm^3+^, NaYF_4_:Yb^3+^, YPO_4_:Eu^3+^, Cs_2_NaYF_6_:Pr^3+^, and ScPO_4_:Gd^3+^ (from top to bottom). **b** Sum of the spectra shown in (**a**). **c** PersL images of NaYF_4_:Pr^3+^, NaYF_4_:Tb^3+^, NaYF_4_:Dy^3+^, NaYF_4_:Ho^3+^, NaYF_4_:Er^3+^ in the visible spectral range. **d** PersL images of NaYF_4_:Nd^3+^, NaYF_4_:Ho^3+^, NaYF_4_:Er^3+^ in the NIR spectral range. **e** PersL decay curve of ScPO_4_:Gd^3+^ at 310 nm. **f** Histograms of PersL intensity of the samples doped with 1% *x*^3+^ (*x* = Ce, Pr, Tb, Dy, Ho, Er, Nd, Sm, Eu, Tm) in NaYF_4_, Cs_2_NaYF_6_, YPO_4_, ScPO_4_, LaPO_4_, and YBO_3_ hosts. Except for YBO_3_ phosphors whose PersL intensity was recorded at 360 s due to the short decay time, the PersL intensity of the rest samples was recorded at an hour after ceasing the radiation of X-ray

Except for La^3+^ without the 4f electrons and Lu^3+^ with full filled 4f shell and radioactive Pm^3+^, the rest trivalent lanthanides’ PersL was observed with success in the selected hosts NaYF_4_, Cs_2_NaYF_6_, YPO_4_, and ScPO_4_ (Fig. 1a, Supplementary Fig. S1). The wavelength of these PersL bands ranges broadly from 200 to 1700 nm (Fig. 1b). The whole wavelength range of the PersL could be divided into the UV, visible, and NIR three parts. Most of the trivalent lanthanides, excluding Gd^3+^ and Yb^3+^, emitted PersL in the visible spectral range. It can be seen that the decay time of the visible PersL is relatively long (Fig. 1c). The shortest decay time originating from the 542 nm emission line of Ho^3+^ and the 542 nm counterpart of Er^3+^ has exceeded 40 h. Notably, the green PersL attributed to the ^5^D_4_ → ^7^F_5_ transition of Tb^3+^ could be collected with a recognizable signal-to-noise ratio even after 200 h. In the wavelength range of 760–1700 nm, Nd^3+^, Ho^3+^, and Er^3+^ emitted the NIR PersL, and their decay time could last for at least 3 h, making these three ions possible luminescent markers for medical imaging (Fig. 1d). The above-mentioned eight PersL bands were clearly measured by spectrometer even after several days (Supplementary Fig. S2). The PersL belonging to the ^6^P_7/2_ → ^8^S_7/2_ transition of Gd^3+^ in ScPO_4_ was also detected directly for the first time, to the best of our knowledge (Fig. 1a, e). It should be mentioned here that Pan’s group has recently also reported the PersL behavior of this transition, which is, however, on the basis of energy transfer from other luminescent centers to Gd^3+^^26^. The decay time of the PersL of Gd^3+^ surpasses 170 h, one of the longest decay times known so far. In addition to Gd^3+^, Pr^3+^ also presented PersL in the UV range peaking at ~251 nm, the shortest wavelength of all the samples reported here (Supplementary Fig. S3).

**Fig. 2:**
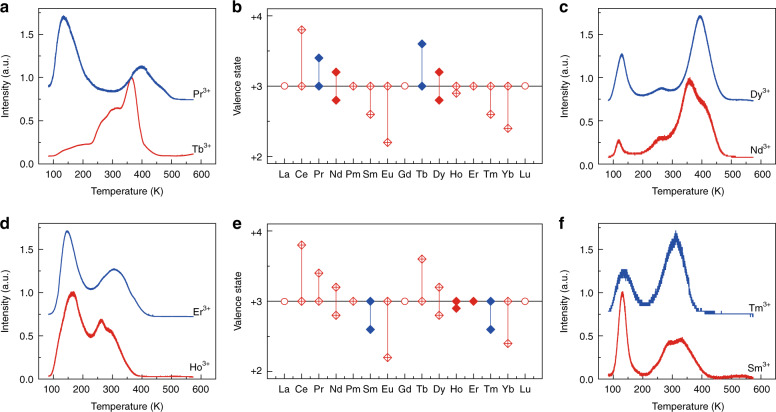
TLs of the trivalent lanthanides in NaYF_4_. **a** TL curves by monitoring the 608 nm line of Pr^3+^ and the 543 nm line of Tb^3+^. **c** TL curves by monitoring the 571 nm line of Dy^3+^ and the 382 nm line of Nd^3+^. **d** TL curves by monitoring the 542 nm line of Er^3+^ and the 542 nm line of Ho^3+^. **f** TL curves by monitoring the 451 nm line of Tm^3+^ and the 594 nm line of Sm^3+^. **b** **e** Variation trend of the valence state of the trivalent lanthanides

After ceasing the X-ray source, the PersL of the trivalent lanthanides in the hosts NaYF_4_, Cs_2_NaYF_6_, YPO_4_, ScPO_4_, LaPO_4_, and YBO_3_ displays different decay behaviors (Fig. 1f, Supplementary Fig. S4–S15). The PersL of Er^3+^, Ho^3+^, and Nd^3+^ is nearly undetectable in the hosts Cs_2_NaYF_6_, YPO_4_, ScPO_4_, LaPO_4_, and YBO_3_, as well as in other hosts such as silicate, aluminate, and zincate (not shown herein). In contrast, Er^3+^, Ho^3+^, and Nd^3+^ emitted considerable PersL that could last for at least 10 h in NaYF_4_. Moreover, the PersL spectra of Er^3+^, Nd^3+^, and Ho^3+^ in Cs_2_NaYF_6_, YPO_4_, ScPO_4_, LaPO_4_, and YBO_3_ are different from that in NaYF_4_ (Supplementary Fig. S16). For instance, Er^3+^ generally emits green and red luminescence which is attributed to the ^2^H_11/2_/^4^S_3/2_ → ^4^I_15/2_ and ^4^F_9/2_ → ^4^I_15/2_ transitions in the 200–800 nm wavelength range, as shown in the top PersL spectrum of Supplementary Fig. S16a for NaYF_4_:Er^3+^^27–29^. Nonetheless, the green and red emissions of Er^3+^ in Cs_2_NaYF_6_, YPO_4_, ScPO_4_, LaPO_4_, and YBO_3_ are seriously weakened. And the transitions with a higher energy than ^2^H_11/2_/^4^S_3/2_ states dominate the emissions. The different emitting characteristics of Er^3+^, Nd^3+^, and Ho^3+^ in different hosts are expected to be responsible for their different PersL behaviors, which will be further discussed in the following. Thermoluminescence (TL) of the prepared samples was also measured (Fig. 2). As can be observed, the positions of the TL peaks of Pr^3+^, Dy^3+^, Er^3+^, and Tm^3+^ correspond roughly to that of Tb^3+^, Nd^3+^, Ho^3+^, and Sm^3+^ (Fig. 2a, c, d, f), which is in good agreement with the variation trend of the valence state of the trivalent lanthanides (Fig. 2b, e)^2^.

## Discussion

### Exciton and attractive potential

The band-to-band excitation is an important and necessary charging way for wide bandgap materials embedded with the trivalent lanthanides^2^. Our samples, due to their wide bandgap, cannot be charged by the excitation source whose energy is below the host’s bandgap such as mercury lamp. As demonstrated before, excitons exist in wide bandgap materials at room temperature as the binding energy of exciton is proportional to the bandgap of host (Supplementary Fig. S17)^30–32^. In order to generate PersL, the trivalent lanthanides, as isoelectronic trap when substituting the trivalent cations in wide bandgap host, are expected to have the ability to bind excitons to get their recombination energy. According to the literature^33^, the ability of binding carriers for the trivalent lanthanides which is defined as the attractive potential *V*, is mainly determined by the intrinsic arrangement of the 4f electrons. Here we show this ability is also associated with the extrinsic ligand field including anion coordination and cation substitution:

1 $$V = V_O + V_A + V_C$$

where *V*_*O*_, *V*_*A*_, and *V*_*C*_ are the attractive potentials aroused by the arrangement of the 4f electrons of the trivalent lanthanides, anion coordination and cation substitution, respectively.

### Intrinsic: arrangement of the 4f electrons

**Fig. 3:**
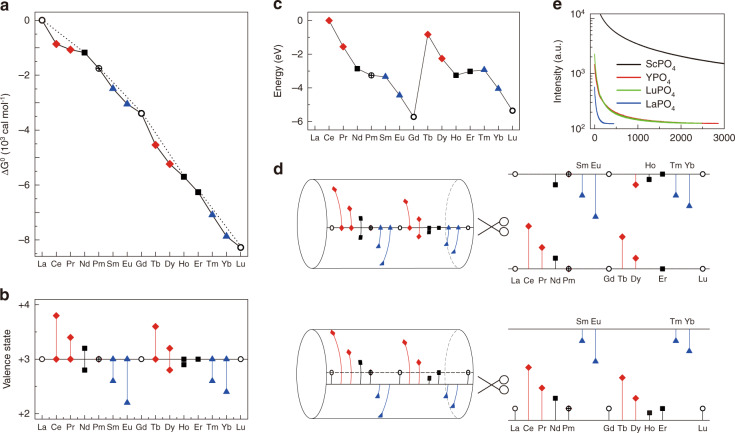
Modulation of the trivalent lanthanides’ attractive potential. **a** Free energy changes (∆G^0^) of the trivalent lanthanides complexes with 2-ethylhexyl phenylphosphonic acid complexing agent relative to that of La^3+^. Reprinted from ref. ^34^, Copyright 1976, with permission from Elsevier. **b** Variation trend of the valence state of the trivalent lanthanides. The length of each vertical line shows the relative ability of changing the valence state. **c** Energy difference between the lowest energy level of the 4f electron configuration of the trivalent lanthanides and that of Ce^3+^. Reprinted from ref. ^35^, Copyright 2009, with permission from Elsevier. **d** Schematic diagram of modulating the position of the trivalent lanthanides in the forbidden band. **e** PersL decay cures of Gd^3+^ doped *X*PO_4_ (*X* = Sc, Y, La, Lu)

This topic is extended herein from three perspectives. First, the double-double effect summarized by Fidelis et al.^34^ indicates that the electron configuration with even total orbital angular momenta is relatively stable (Supplementary Table S1), including La^3+^, Gd^3+^, Lu^3+^, Er^3+^, Nd^3+^, Ho^3+^, Pm^3+^. By comparison, the trivalent lanthanides with odd total orbital angular momenta, that are, Ce^3+^, Tb^3+^, Pr^3+^, Dy^3+^, Eu^3+^, Sm^3+^, Tm^3+^, and Yb^3+^, are not such stable. It reveals that the stability of Er^3+^, Nd^3+^, Ho^3+^, and Pm^3+^ is close to that of La^3+^, Gd^3+^, and Lu^3+^. As clearly presented in Fig. 3a, the whole trivalent lanthanides can be grouped into [Ce^3+^, Pr^3+^, Tb^3+^, Dy^3+^, Sm^3+^, Eu^3+^, Tm^3+^, and Yb^3+^] and [Er^3+^, Nd^3+^, Ho^3+^, Pm^3+^, La^3+^, Gd^3+^, and Lu^3+^], which agrees well with the order of stability. Second, the variation trend of valence state suggests obviously that La^3+^, Gd^3+^, and Lu^3+^ are the most stable trivalent lanthanides, followed by Er^3+^, Nd^3+^, Ho^3+^ and Pm^3+^ (Fig. 3b)^2^. Ce^3+^, Pr^3+^, Tb^3+^, and Dy^3+^ are inclined to lose electron to be quadrivalent while Sm^3+^, Eu^3+^, Tm^3+^, and Yb^3+^ tend to gain electron to be bivalent. It can be concluded that the result in Fig. 3b is almost identical to the law presented in Fig. 3a. The major difference is that the double-double effect is unidirectional while the variation trend of valence state is bidirectional. So the undetectable PersL of Er^3+^, Nd^3+^, and Ho^3+^ in oxysalts is explained by the fact that it is difficult for them to bind carries (Fig. 1f). The similar TL curves (Fig. 2a, c, d, f) of four groups of the trivalent lanthanides could also be reasonably ascribed to the similar carrier-binding ability. Third, the energy difference between the lowest energy level of the 4f electron configuration of the trivalent lanthanides and that of Ce^3+^ shows the similar results with the above-mentioned two evidences (Fig. 3c)^35^. On the whole, the trivalent lanthanides can be roughly divided into four groups, [Ce^3+^, Pr^3+^, Tb^3+^, Dy^3+^], [Sm^3+^, Eu^3+^, Tm^3+^, Yb^3+^], [Er^3+^, Nd^3+^, Ho^3+^, Pm^3+^], and [La^3+^, Gd^3+^, Lu^3+^], although there might be some little discrepancy.

All these evidences indicate undoubtedly that the attractive potential of the trivalent lanthanides at their free state is mainly determined by the intrinsic arrangement of the 4f electrons, which gives us a clear figure of the ability of binding carriers for the trivalent lanthanides in wide bandgap materials. It’s worth noting that the zig-zag model cannot be used to explain our findings here, especially the ultralong PersL of phosphors doped with Gd^3+^. Gd is always in +3 valence outside of several exotic compounds. Therefore, we cannot use the core point of the zig-zag model, i.e., valence alternation, to account for the ultralong PersL of phosphors doped with Gd^3+^. More importantly, the ground state of Gd^2+^ is always above the conduction band edge and that of Gd^3+^ will be firmly below the valence band edge, and the so-called “bandgap engineering” does not change the fact. In light of the zig-zag model, the PersL of phosphors doped with Gd^3+^ should never occur, which is obviously against our findings. So the zig-zag model is not the substance of the PersL of the trivalent lanthanides in wide bandgap materials, although it sometimes reflects similar trends. At this point, it should be emphasized that the above discussions are only suitable for free trivalent lanthanides, meaning that they only reflect the property of the intrinsic arrangement of the 4f electrons. In addition to this “internal” factor, the “external” aspects, mainly including anion coordination and cation substitution, have also an effect on the ability of binding carriers of the trivalent lanthanides in crystals, as discussed below.

### Extrinsic I: anion coordination

In ionic crystals such as NaYF_4_, the valence electrons of the trivalent lanthanides are completely occupied by the coordination anions. The attractive potential of the trivalent lanthanides’ 4f electrons is thus less affected by the shielding effect of the valence electrons. The effect of electron cloud polarization becomes appreciable, making there an additional ability on the original attractive potential of the trivalent lanthanides’ 4f electrons. Therefore, Er^3+^, Ho^3+^, and Nd^3+^ that are not easy to bind charges also own the ability to bind carriers to generate emission upon band-to-band excitation. In phosphates and borates hosts, the interaction between the trivalent lanthanides and coordination anions changes from ionic to covalent. The increase in the electrostatic shielding effect of valence electrons on the trivalent lanthanides’ 4f electrons results in a decrease of their attractive potential. As mentioned above, Er^3+^, Ho^3+^, and Nd^3+^ in their free state have weak ability to bind charge carriers, and this ability has been further weakened in covalent crystals. This probably explains the missing PersL of Er^3+^, Ho^3+^, and Nd^3+^ in phosphates and borates (Fig. 1f).

### Extrinsic II: cation substitution

In addition to the strategy of anion coordination, cation substitution is also commonly utilized to adjust crystalline field. When substituting the trivalent lanthanides for the trivalent host cations, the isoelectronic traps are formed, followed by the generation of bound state by a short-range central-cell potential. According to the reported work^36–39^, the primary factor affecting the binding potential of the isoelectronic traps is electronegativity and size difference between the impurity and host ions. Therefore, the difference of electronegativity and the size difference between the substitution ion and the trivalent lanthanides make there a bound state for the trivalent lanthanides in the forbidden band. The attractive potential and position of the trivalent lanthanides in the forbidden band are thus adjustable by changing the cation substitution (Fig. 3d). It reminds us that the PersL of the trivalent lanthanides can be expected, adjusted, and even improved via a rational design of the external factor of cation substitution. It has been verified by the samples of Gd^3+^ doped *X*PO_4_ (*X* = Sc, Y, La, Lu) (Fig. 3e). The longest PersL of Gd^3+^ is achieved in ScPO_4_ because Sc^3+^, compared with Lu^3+^, Y^3+^, and La^3+^, has a larger difference in electronegativity and size with Gd^3+^. The similar conclusion was also observed in the samples of Gd^3+^ doped *X*BO_3_ (*X* = Sc, Y, La, Lu) (Supplementary Fig. S18). The larger the difference between Gd^3+^ and substitution ion, the longer the PersL decay time of Gd^3+^, in good accordance with the above-mentioned hypothesis.

### PersL mechanism of the trivalent lanthanides

**Fig. 4:**
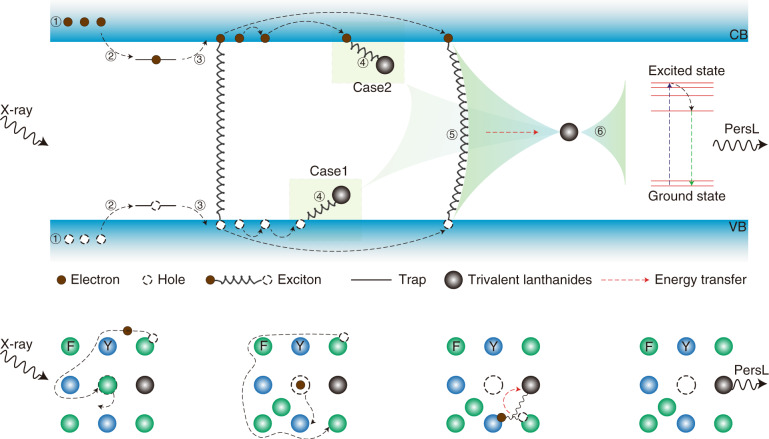
Mechanism of the PersL of the trivalent lanthanides in wide bandgap hosts. The top panel shows the possible PersL processes depending on the commonly used valence band (VB) and conduction band (CB) model. The bottom panel shows the possible PersL processes at the atomic level, by taking the host NaYF_4_ doped with the trivalent lanthanides as an example

Depending on the above-mentioned discussions, the possible mechanism of the trivalent lanthanides’ PersL in wide bandgap hosts is proposed (top panel in Fig. 4). Upon excitation of X-ray, the electrons jump from the valence band to the conduction band, leaving there a large amount of holes in the valence band (①). Subsequently, these electrons and holes are captured by the traps (②). After ceasing the X-ray source, the captured electrons and holes are released from the traps to the conduction band and valence band, respectively, due to the stimulation of heat (③). The trivalent lanthanides attract an electron (or hole) first and then draw a hole (or electron) due to the Coulomb force (④, ⑤, case 1 and case 2). It should be emphasized there is the possibility that the released electrons and holes attract each other to form excitons. Due to the electrically neutral characteristic, the excitons migrate among the crystal lattice and are finally captured by the trivalent lanthanides (⑤). In all cases, the trivalent lanthanides are expected to finally bound the excitons and then obtain the recombination energy of the bounded excitons to jump to the excited states (⑥)^11^. After non-radiative relaxation processes, the PersL occurs due to the 4f ↔ 4f transition of the trivalent lanthanides (⑥).

To further understand the specific generation process of PersL, the possible mechanism is then discussed at the atomic level (bottom panel in Fig. 4), by taking the host NaYF_4_ embedded with the trivalent lanthanides as an example. For NaYF_4_, the top of the valence band and the bottom of the conduction band separately belong to the 2p electron orbit of F and the 5d electron orbit of Y. The isoelectronic cation substitution, namely replacing the Y^3+^ in NaYF_4_ with other trivalent lanthanides, forms bound states by a short-range central-cell potential. Upon excitation of X-ray, the outer electrons of F are excited to the 5d electron orbit of Y, while the generated holes stay in F. Upon X-ray irradiation, the F ions are likely to be ejected from their original sites, making there many fluorine vacancies and interstitial F that act as traps^29^. The electrons in the conduction band and the holes in the valence band are thus trapped with success. After ceasing the X-ray source, the electrons are released from the defects to the 5d electron orbit of Y due to the stimulation of heat. The trivalent lanthanides are expected to attract an electron (or hole) first and then will draw a hole (or electron) due to the Coulomb force. It should be mentioned that the released electrons and holes are likely to attract each other to form excitons that migrate easily among the crystal lattice because of the electrically neutral feature. In all cases, the trivalent lanthanides eventually bind the excitons as this is the origin of energy. The excitons recombine and transfer the energy to the trivalent lanthanides that bind the excitons. The trivalent lanthanides are excited from the ground state to the excited state, which is followed by the non-radiative relaxation processes and final emitting of PersL.

## Conclusion

In summary, the PersL of the trivalent lanthanides with the exception of Lu^3+^, La^3+^, and Pm^3+^ covering a broad wavelength range from 200 to 1700 nm are observed in several wide bandgap materials, upon the band-to-band charging way via X-ray irradiation. A mechanism of the trivalent lanthanides’ PersL in wide bandgap hosts is proposed. According to the mechanism, the trivalent lanthanides as isoelectronic traps are expected to eventually bind excitons, and this binding ability is not only related to the inherent arrangement of the 4f electrons of the trivalent lanthanides, but also to the extrinsic anion coordination and cation substitution. The excitons in wide bandgap materials transfer their recombination energy to the trivalent lanthanides, followed by the generation of PersL from the trivalent lanthanides. Guided by this mechanism, the direct ultralong PersL of Gd^3+^ is achieved for the first time in phosphates, proving the validity of our proposed mechanism. Our work not only widens the range of the trivalent lanthanides activated PersL phosphors but also proposes a mechanism with considerable rationality, which is believed to be a guidance for designing high-performance and more ideal PersL phosphors in the future.

## Materials and methods

### Fabrication of samples

All PersL phosphors were prepared by high-temperature solid-state method and the doping concentration of the trivalent lanthanides was set to be 1% mol. NaF (Aladdin, 99.99%), NH_4_F (Aladdin, 99.99%), *X*_2_O_3_ (*X* = Y, La, Ce, Nd, Sm, Eu, Gd, Dy, Ho, Er, Tm, and Yb, Aladdin, 99.99%), Pr_6_O_11_ (Aladdin, 99.99%), Tb_4_O_7_ (Aladdin, 99.99%), Cs_2_CO_3_ (Aladdin, 99.99%), NaHCO_3_ (Aladdin, 99.99%), NH_4_H_2_PO_4_ (Aladdin, 99.95%), Sc_2_O_3_ (Aladdin, 99.99%), and H_3_BO_3_ (Aladdin, 99.99%) were used as raw materials. The general preparation processes for the samples are described as follows. The stoichiometric raw materials were weighed and mixed well in mortar to form homogeneous powders. These powders were then transferred into aluminum oxide crucible to be calcined in muffle furnace at the given temperature for several hours. Finally, the obtained powders were cooled down to room temperature to form the final phosphors. To form NaYF_4_ phosphors, the homogeneous powders were calcined at 500 °C for 2 h. For the preparation of Cs_2_NaYF_6_, the homogeneous powders were pre-fired at 150 °C in air for 7 h, and were then sintered at 450 °C for 30 min, followed by the final calcination at 700 °C for 10 h under nitrogen atmosphere. To obtain YPO_4_, ScPO_4_, and LaPO_4_ samples, the homogeneous raw materials should be calcined at 500 °C for 2 h under nitrogen atmosphere first and then at 1300 °C for 5 h. To get YBO_3_ phosphors, the raw powders were annealed under nitrogen atmosphere at 500 °C for 1 h first and then at 1300 °C for 2 h. It was demonstrated that the samples were micro-sized with irregular shape (Supplementary Figs. S19–S24). In addition, these phosphors were of pure phase (Supplementary Fig. S25).

### Charging of samples

The PersL phosphors were charged by the X-ray source of XRad-320X-ray irradiator (Precision X-ray, Inc., North Branford, CT) equipped with a tungsten target (40 kV, 30 mA). Before measurement on the property of PersL, all samples were irradiated by X-ray for 10 min to be charged.

### Photoluminescence (PL) and PersL characterization of samples

The PL spectra of samples upon X-ray excitation, PersL spectra, and decay curves of PersL in the visible light band were recorded using an Andor SR-500i spectrometer (Andor Technology Co. Belfast, UK) equipped with a Hamamatsu R928 photomultiplier. The visible PersL images were recorded using a digital SLR camera (EOS 5D Mark III) in darkroom. The NIR PersL images were recorded using a CCD camera (DU-888U3-CS0-BV).

### TL characterization of samples

TL measurements were carried out using a self-assembling TL system including high precision thermal stage (THMS600) (British Linkam Scientific Instruments) and a Andor SR-500i spectrometer (Andor Technology Co., Belfast, UK), with a fixed heating rate of 5 K/s within the range of 83–600 K. The samples were irradiated by X-ray for 10 min before TL measurement.

### Vacuum ultraviolet (VUV) excitation characterization of samples

The VUV excitation spectra were measured at the VUV spectroscopy experimental station on beam line 4B8 of Beijing Synchrotron Radiation Facility (BSRF). The excitation and emission spectra were measured by using a 1 m Seya monochromator (1200 g·mm^−1^, 120–350 nm, 1 nm bandwidth) and an Acton SP-308 monochromator (600 g·mm^−1^, 330–900 nm). The signal was detected by a Hamamatsu H8259-01 photon-counting unit and corrected by the excitation intensity of sodium salicylate (o-C_6_H_4_OHCOONa) measured simultaneously under the same condition.

## Supplementary information

Supplementary File
