# Supplementary material for: Mechanism of the trivalent lanthanides’ persistent luminescence in wide bandgap materials
Source: Light Sci Appl. 2022 Mar 8;11:51. doi: 10.1038/s41377-022-00736-5 (PMC8901650; doi:10.1038/s41377-022-00736-5)
Supplement: Supplementary file 1 — Supplementary File [file 41377_2022_736_MOESM1_ESM.pdf]

Supplementary Information for

Mechanism of the trivalent lanthanides' persistent luminescence in wide bandgap materials

Leipeng Li+, Tianyi Li+, Yue Hu+, Chongyang Cai+, Yunqian Li, Xuefeng Zhang, Baolai Liang, Yanmin Yang* and Jianrong Qiu*

Dr. L. Li, T. Li, Y. Hu, C. Cai, Y. Li, X. Zhang, Prof. B. Liang, Prof. Y. Yang
Hebei Key Laboratory of Optic-electronic Information and Materials, College of Physics Science & Technology, Hebei University, Baoding 071002, China
E-mail: yangym@hbu.edu.cn (Y. Yang)

Prof. B. Liang
California NanoSystems Institute, University of California - Los Angeles, CA 90095, USA

Prof. J. Qiu
State Key Laboratory of Modern Optical Instrumentation, College of Optical Science and Engineering, Zhejiang University, Hangzhou 310058, China
E-mail: qjr@zju.edu.cn

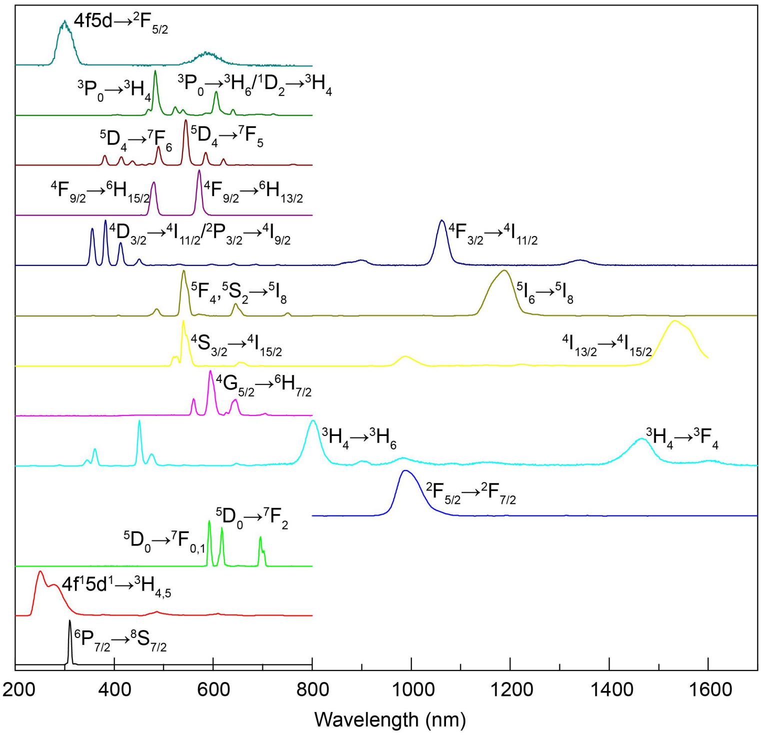

Figure S1. Normalized PersL spectra of $NaYF_4$:Ce^{3+}, $NaYF_4$:Pr^{3+}, $NaYF_4$:Tb^{3+}, $NaYF_4$:Dy^{3+}, $NaYF_4$:Nd^{3+}, $NaYF_4$:Ho^{3+}, $NaYF_4$:Er^{3+}, $NaYF_4$:Sm^{3+}, $NaYF_4$:Tm^{3+}, $NaYF_4$:Yb^{3+}, YPO_4:Eu^{3+}, Cs_2NaYF_6:Pr^{3+} and $ScPO_4$:Gd^{3+} (from top to bottom). The affiliations of the main PersL bands of each spectrum have been marked clearly.

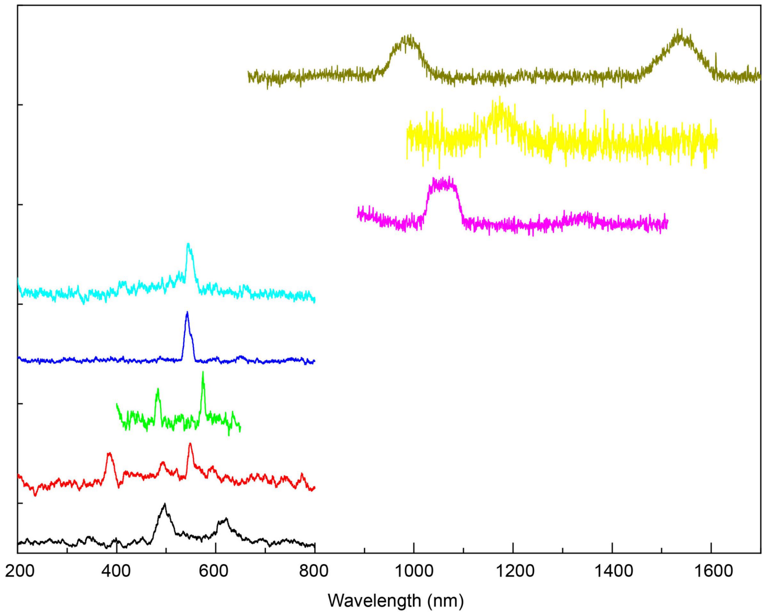

Figure S2. Normalized PersL spectra of $NaYF_4$ doped with different trivalent lanthanides after long decay time. These spectra from top to bottom were measured after 50 h ($NaYF_4$:Er^{3+}), 35 h ($NaYF_4$:Ho^{3+}), 80 h ($NaYF_4$:Nd^{3+}), 40 h ($NaYF_4$:Er^{3+}), 40 h ($NaYF_4$:Ho^{3+}), 50 h ($NaYF_4$:Dy^{3+}), 120 h ($NaYF_4$:Tb^{3+}) and 80 h ($NaYF_4$:Pr^{3+}), respectively.

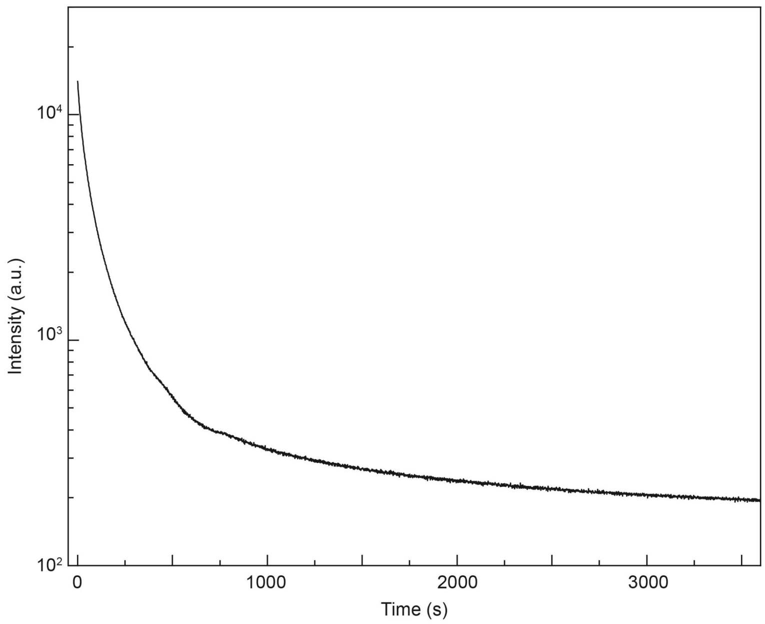

Figure S3. PersL decay curve of Cs_2NaYF_6:Pr^{3+} monitored at 251 nm.

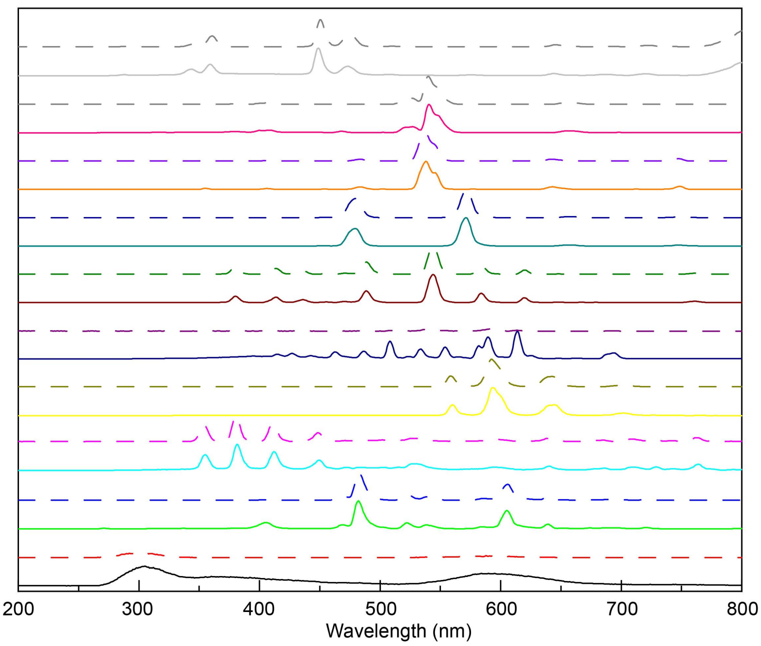

Figure S4. The dotted and solid lines show the normalized PersL and PL spectra of $NaYF_4$ samples doped with different trivalent lanthanides, respectively. Each PersL spectrum and the adjacent lower PL spectrum are a group of spectra from the same sample. The spectra groups from top to bottom come from $NaYF_4:Tm^{3+}$, $NaYF_4:Er^{3+}$, $NaYF_4:Ho^{3+}$, $NaYF_4:Dy^{3+}$, $NaYF_4:Tb^{3+}$, $NaYF_4:Eu^{3+}$, $NaYF_4:Sm^{3+}$, $NaYF_4:Nd^{3+}$, $NaYF_4:Pr^{3+}$ and $NaYF_4:Ce^{3+}$, respectively.

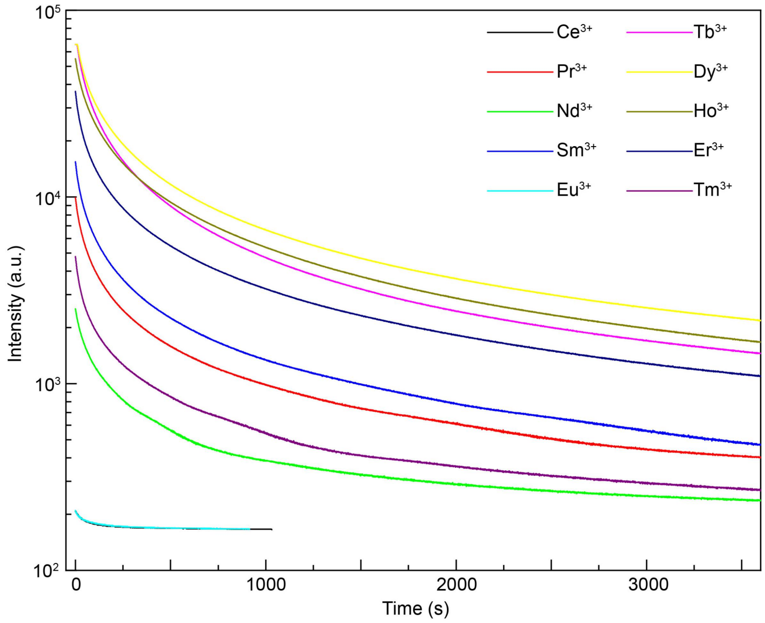

Figure S5. Decay curves of the trivalent lanthanides' PersL in $NaYF_4$ after ceasing the X-ray excitation.

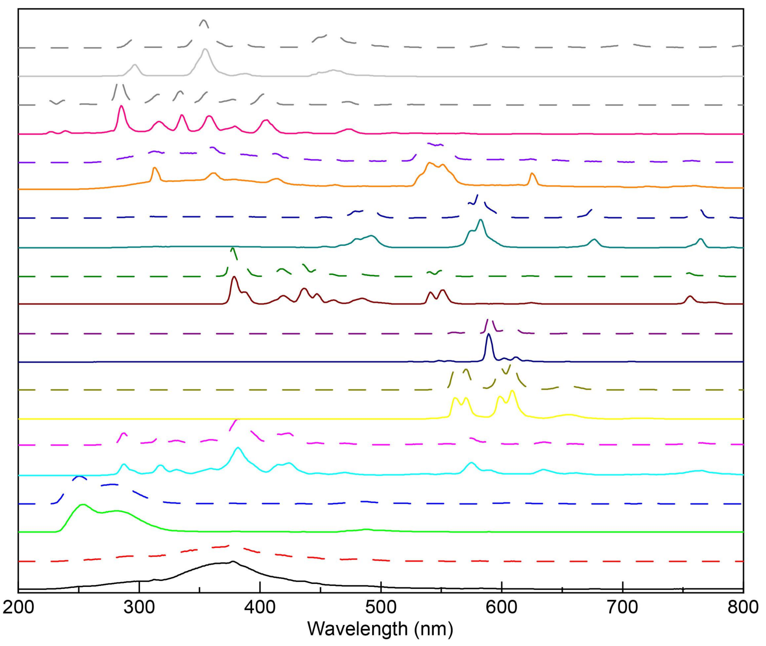

Figure S6. The dotted and solid lines show the normalized PersL and PL spectra of Cs_2NaYF_6 samples doped with different trivalent lanthanides, respectively. Each PersL spectrum and the adjacent lower PL spectrum are a group of spectra from the same sample. The spectra groups from top to bottom come from Cs_2NaYF_6:Tm^{3+}, Cs_2NaYF_6:Er^{3+}, Cs_2NaYF_6:Ho^{3+}, Cs_2NaYF_6:Dy^{3+}, Cs_2NaYF_6:Tb^{3+}, Cs_2NaYF_6:Eu^{3+}, Cs_2NaYF_6:Sm^{3+}, Cs_2NaYF_6:Nd^{3+}, Cs_2NaYF_6:Pr^{3+} and Cs_2NaYF_6:Ce^{3+}, respectively.

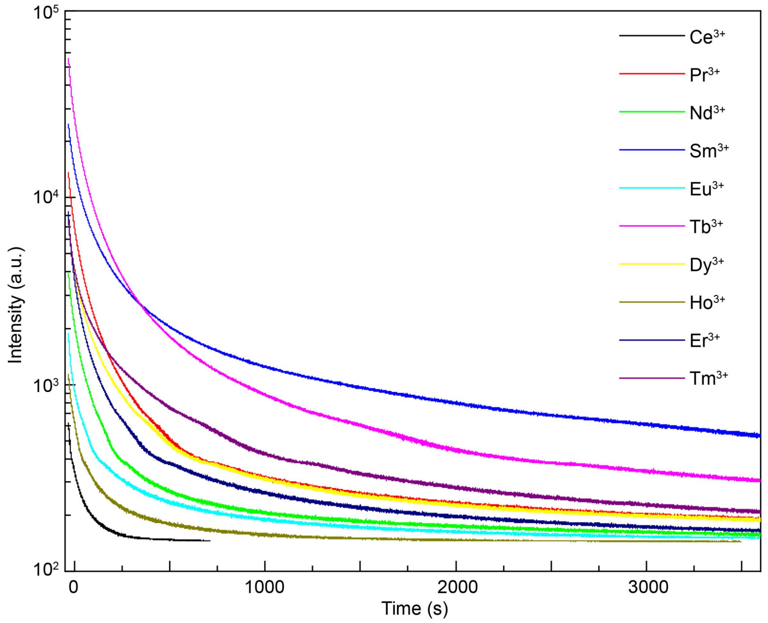

Figure S7. Decay curves of the trivalent lanthanides' PersL in Cs_2NaYF_6 after ceasing the X-ray excitation.

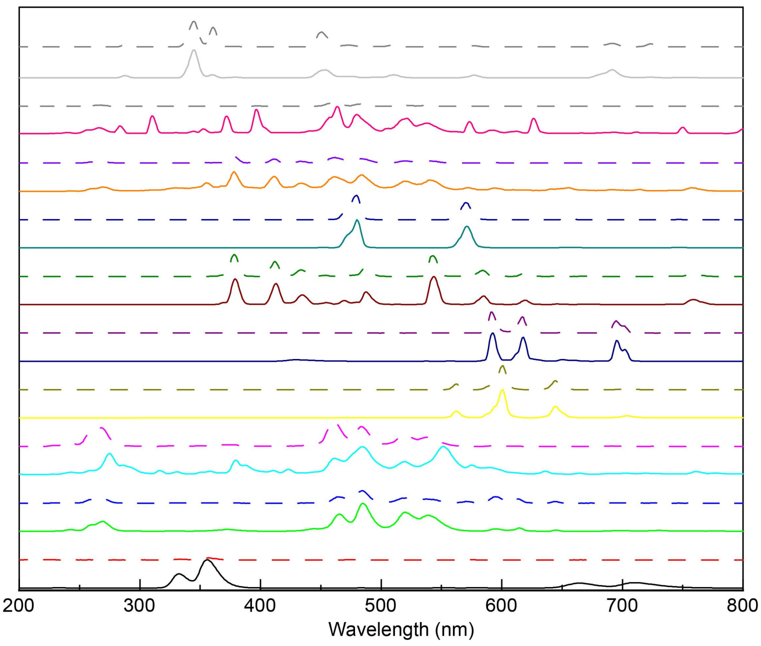

Figure S8. The dotted and solid lines show the normalized PersL and PL spectra of YPO_4 samples doped with different trivalent lanthanides, respectively. Each PersL spectrum and the adjacent lower PL spectrum are a group of spectra from the same sample. The spectra groups from top to bottom come from YPO_4:Tm^{3+}, YPO_4:Er^{3+}, YPO_4:Ho^{3+}, YPO_4:Dy^{3+}, YPO_4:Tb^{3+}, YPO_4:Eu^{3+}, YPO_4:Sm^{3+}, YPO_4:Nd^{3+}, YPO_4:Pr^{3+} and YPO_4:Ce^{3+}, respectively.

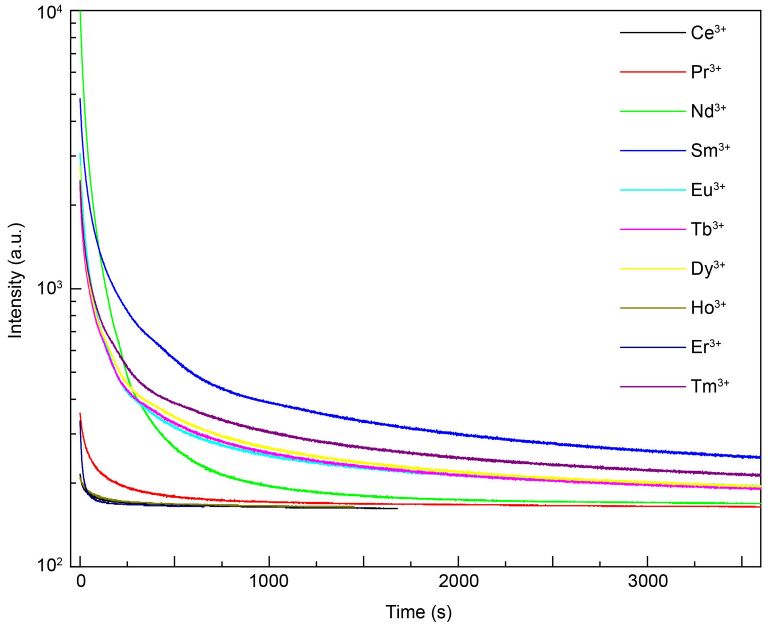

Figure S9. Decay curves of the trivalent lanthanides' PersL in YPO_4 after ceasing the X-ray excitation.

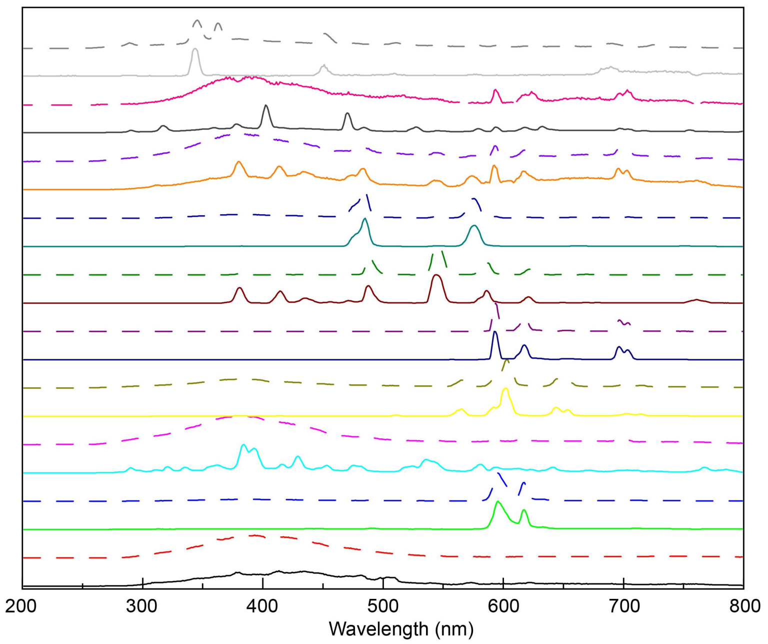

Figure S10. The dotted and solid lines show the normalized PersL and PL spectra of $ScPO_4$ samples doped with different trivalent lanthanides, respectively. Each PersL spectrum and the adjacent lower PL spectrum are a group of spectra from the same sample. The spectra groups from top to bottom come from $ScPO_4$:Tm^{3+}, $ScPO_4$:Er^{3+}, $ScPO_4$:Ho^{3+}, $ScPO_4$:Dy^{3+}, $ScPO_4$:Tb^{3+}, $ScPO_4$:Eu^{3+}, $ScPO_4$:Sm^{3+}, $ScPO_4$:Nd^{3+}, $ScPO_4$:Pr^{3+} and $ScPO_4$:Ce^{3+}, respectively.

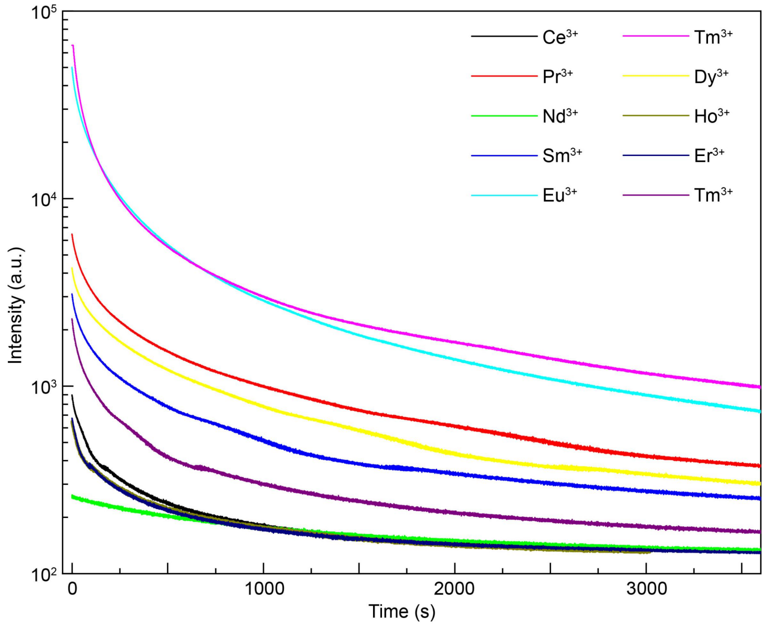

Figure S11. Decay curves of the trivalent lanthanides' PersL in $ScPO_4$ after ceasing the X-ray excitation.

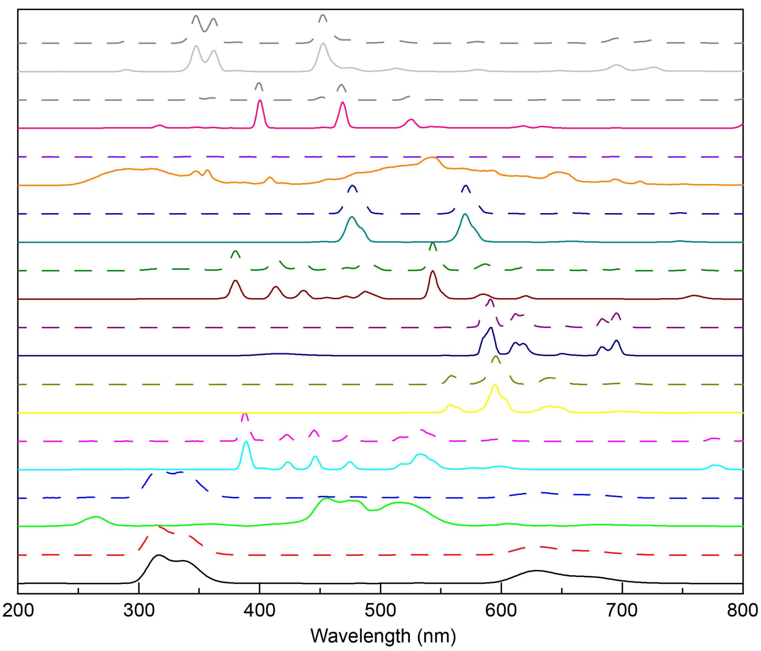

Figure S12. The dotted and solid lines show the normalized PersL and PL spectra of $LaPO_4$ samples doped with different trivalent lanthanides, respectively. Each PersL spectrum and the adjacent lower PL spectrum are a group of spectra from the same sample. The spectra groups from top to bottom come from $LaPO_4:Tm^{3+}$, $LaPO_4:Er^{3+}$, $LaPO_4:Ho^{3+}$, $LaPO_4:Dy^{3+}$, $LaPO_4:Tb^{3+}$, $LaPO_4:Eu^{3+}$, $LaPO_4:Sm^{3+}$, $LaPO_4:Nd^{3+}$, $LaPO_4:Pr^{3+}$ and $LaPO_4:Ce^{3+}$, respectively.

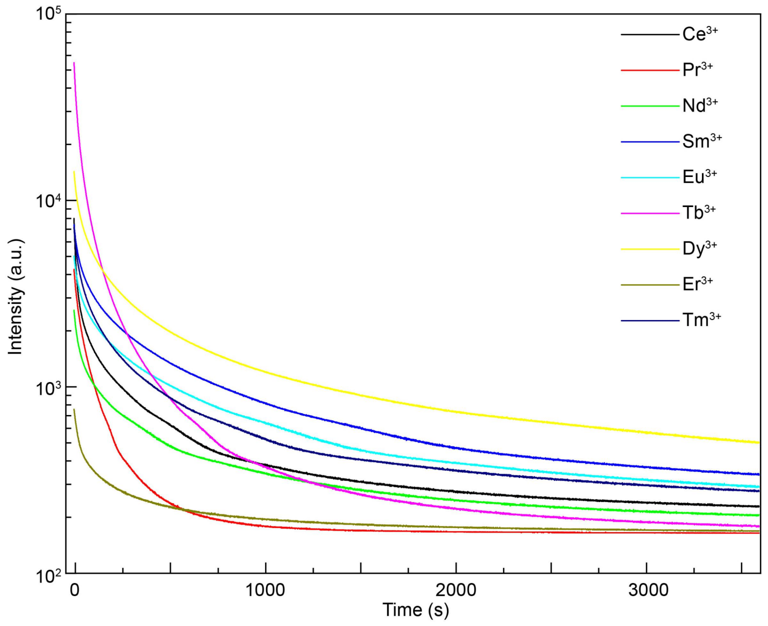

Figure S13. Decay curves of the trivalent lanthanides' PersL in $LaPO_4$ after ceasing the X-ray excitation.

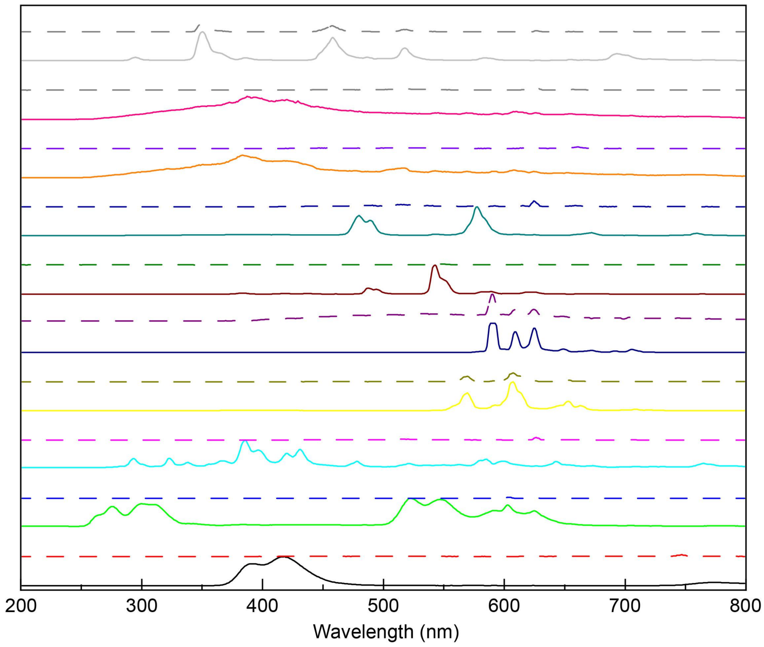

Figure S14. The dotted and solid lines show the normalized PersL and PL spectra of YBO_3 samples doped with different trivalent lanthanides, respectively. Each PersL spectrum and the adjacent lower PL spectrum are a group of spectra from the same sample. The spectra groups from top to bottom come from $YBO_3:Tm^{3+}$, $YBO_3:Er^{3+}$, $YBO_3:Ho^{3+}$, $YBO_3:Dy^{3+}$, $YBO_3:Tb^{3+}$, $YBO_3:Eu^{3+}$, $YBO_3:Sm^{3+}$, $YBO_3:Nd^{3+}$, $YBO_3:Pr^{3+}$ and $YBO_3:Ce^{3+}$, respectively.

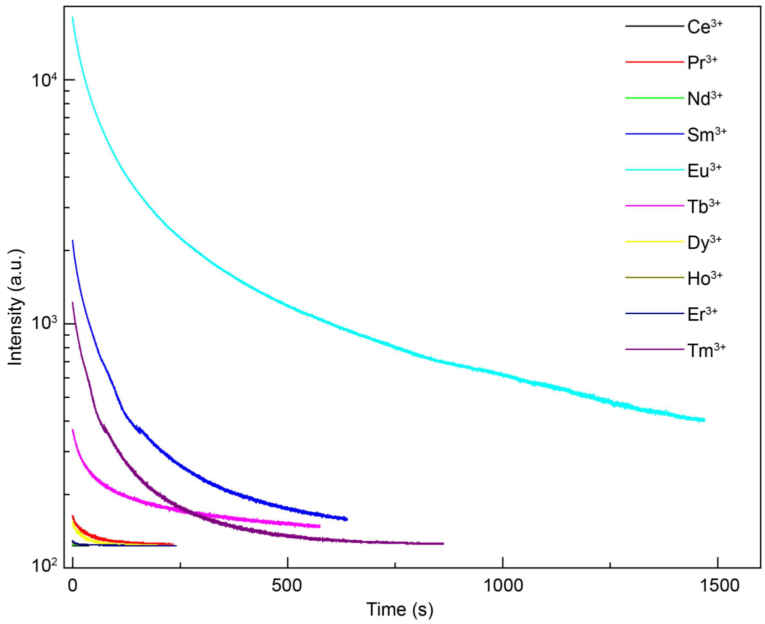

Figure S15. Decay curves of the trivalent lanthanides' PersL in YBO_3 after ceasing the X-ray excitation.

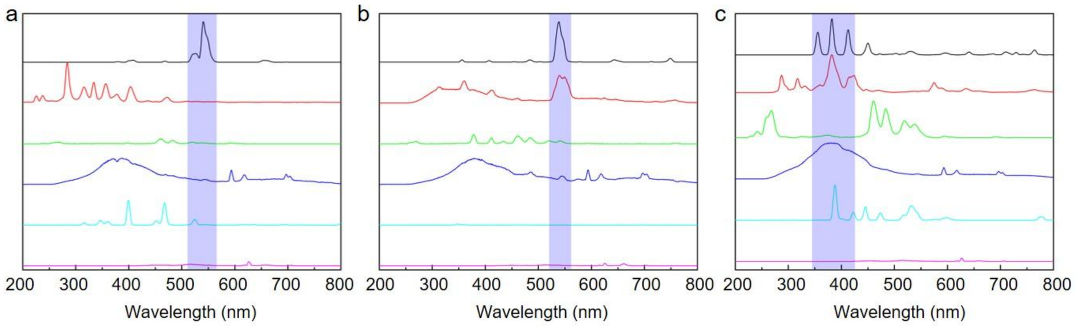

Figure S16. PersL spectra of **(a)** Er^{3+} **(b)** Ho^{3+} and **(c)** Nd^{3+} in different hosts. For each panel, the hosts from top to bottom are $NaYF_4$, Cs_2NaYF_6, YPO_4, $ScPO_4$, $LaPO_4$ and YBO_3, respectively.

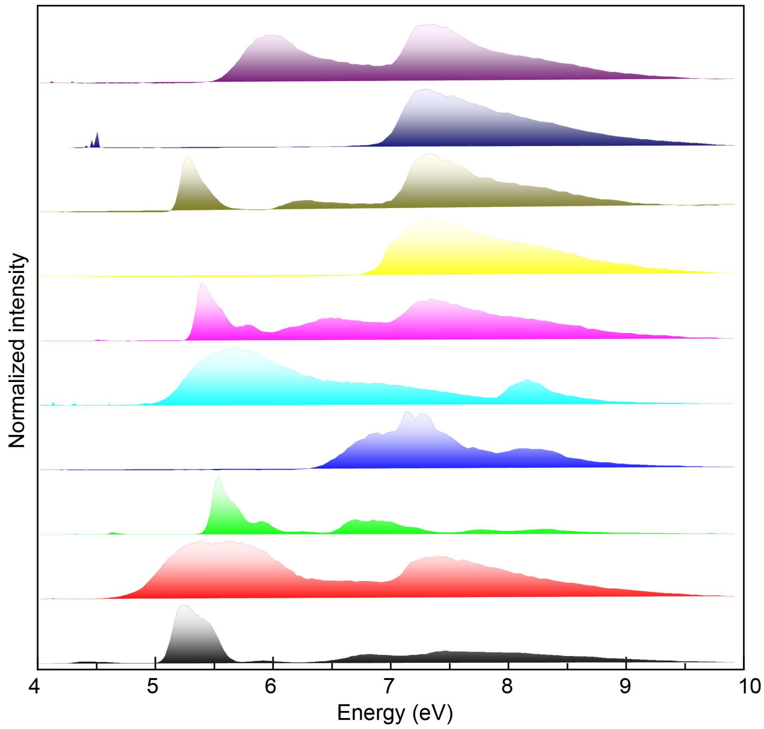

Figure S17. Room temperature PLE spectra from top to bottom separately come from $ScPO_4:Eu^{3+}$, $ScPO_4:Gd^{3+}$, $ScPO_4:Pr^{3+}$, $ScPO_4:Sm^{3+}$, $ScPO_4:Tb^{3+}$, $YPO_4:Eu^{3+}$, $YPO_4:Sm^{3+}$, $YPO_4:Tb^{3+}$, $YBO_3:Eu^{3+}$ and $YBO_3:Tb^{3+}$, by monitoring the emissions of Eu^{3+} at 594 nm, Gd^{3+} at 310 nm, Pr^{3+} at 596 nm, Sm^{3+} at 603 nm, and Tb^{3+} at 544 nm. The broad excitation bands at ~7.5 eV for $ScPO_4$, at ~8.2 eV for YPO_4 and at ~7.3 eV for YBO_3 are attributed to the absorption of the hosts.[1,2] The presence of these excitation bands is indicative of the energy transfer from hosts (excitons) to the trivalent lanthanides.

Table S1. Comparison of spectra terms, total orbital angular momenta and relative stability of $La^{3+}(4f^0)$, $Ce^{3+}(4f^1)$, $Pr^{3+}(4f^2)$, $Nd^{3+}(4f^3)$, $Pm^{3+}(4f^4)$, $Sm^{3+}(4f^5)$, $Eu^{3+}(4f^6)$, $Gd^{3+}(4f^7)$, $Tb^{3+}(4f^8)$, $Dy^{3+}(4f^9)$, $Ho^{3+}(4f^{10})$, $Er^{3+}(4f^{11})$, $Tm^{3+}(4f^{12})$, $Yb^{3+}(4f^{13})$ and $Lu^{3+}(4f^{14})$.

**The trivalent lanthanides**	**Designated letter for *L***	**Total orbital angular momenta *L***	**Relative stability**
$Ce^{3+}(4f^1)$, $Eu^{3+}(4f^6)$, $Tb^{3+}(4f^8)$, $Yb^{3+}(4f^{13})$	F	3	Low
$Pr^{3+}(4f^2)$, $Sm^{3+}(4f^5)$, $Dy^{3+}(4f^9)$ $Tm^{3+}(4f^{12})$	H	5	Moderate
$Nd^{3+}(4f^3)$, $Pm^{3+}(4f^4)$, $Ho^{3+}(4f^{10})$ $Er^{3+}(4f^{11})$	I	6	Relatively high
$La^{3+}(4f^0)$, $Gd^{3+}(4f^7)$ $Lu^{3+}(4f^{14})$	S	0	High

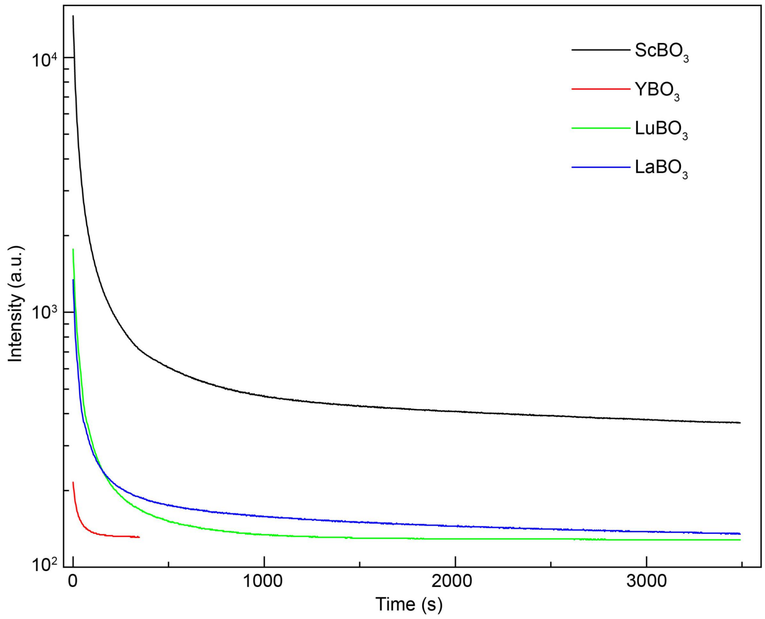

Figure S18. PersL decay curves of Gd^{3+} doped *X*BO_3 (*X* = Sc, Y, La, Lu).

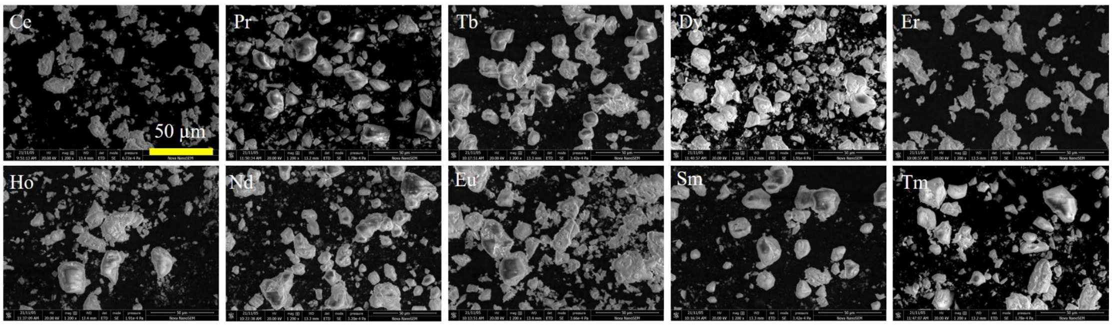

Figure S19. SEM images of Cs_2NaYF_6:X^{3+} (X = Ce, Pr, Tb, Dy, Er, Ho, Nd, Eu, Sm and Tm)

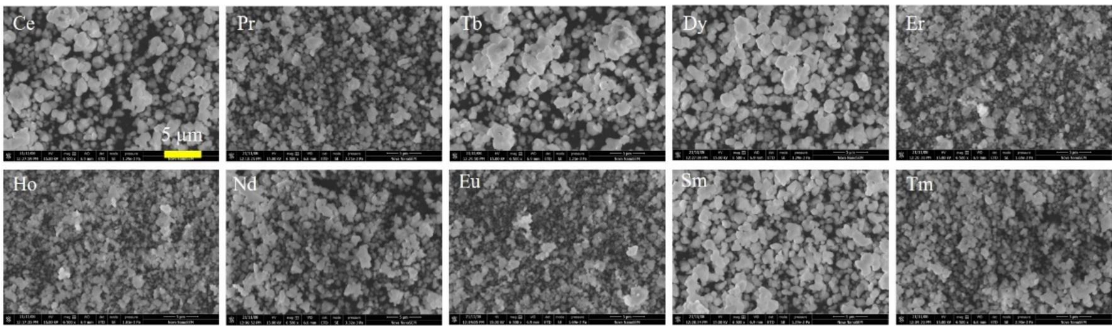

Figure S20. SEM images of $NaYF_4$:X^{3+} (X = Ce, Pr, Tb, Dy, Er, Ho, Nd, Eu, Sm and Tm)

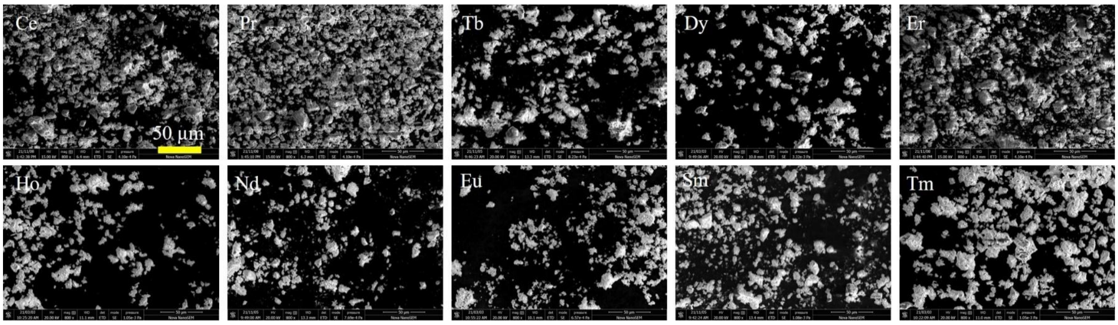

Figure S21. SEM images of YPO_4:X^{3+} (X = Ce, Pr, Tb, Dy, Er, Ho, Nd, Eu, Sm and Tm)

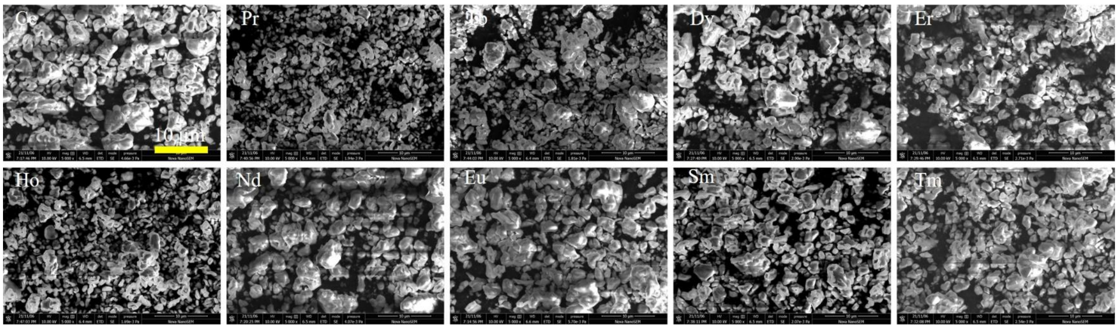

Figure S22. SEM images of $ScPO_4$:X^{3+} (X = Ce, Pr, Tb, Dy, Er, Ho, Nd, Eu, Sm and Tm)

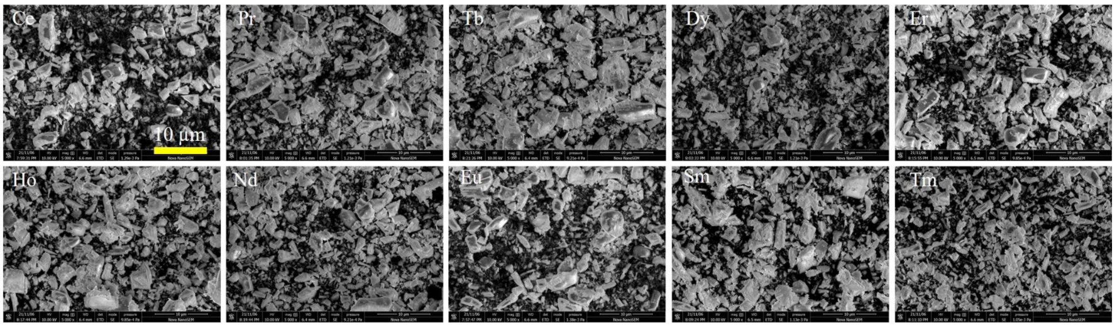

Figure S23. SEM images of $LaPO_4$:X^{3+} (X = Ce, Pr, Tb, Dy, Er, Ho, Nd, Eu, Sm and Tm)

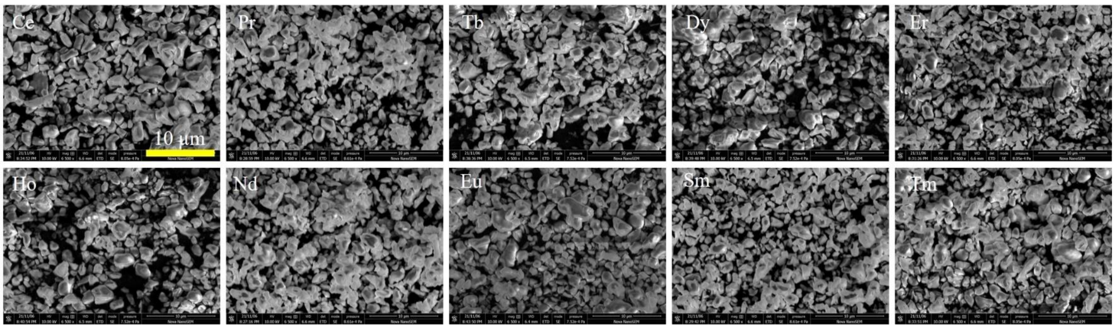

Figure S24. SEM images of YBO_3:X^{3+} (X = Ce, Pr, Tb, Dy, Er, Ho, Nd, Eu, Sm and Tm)

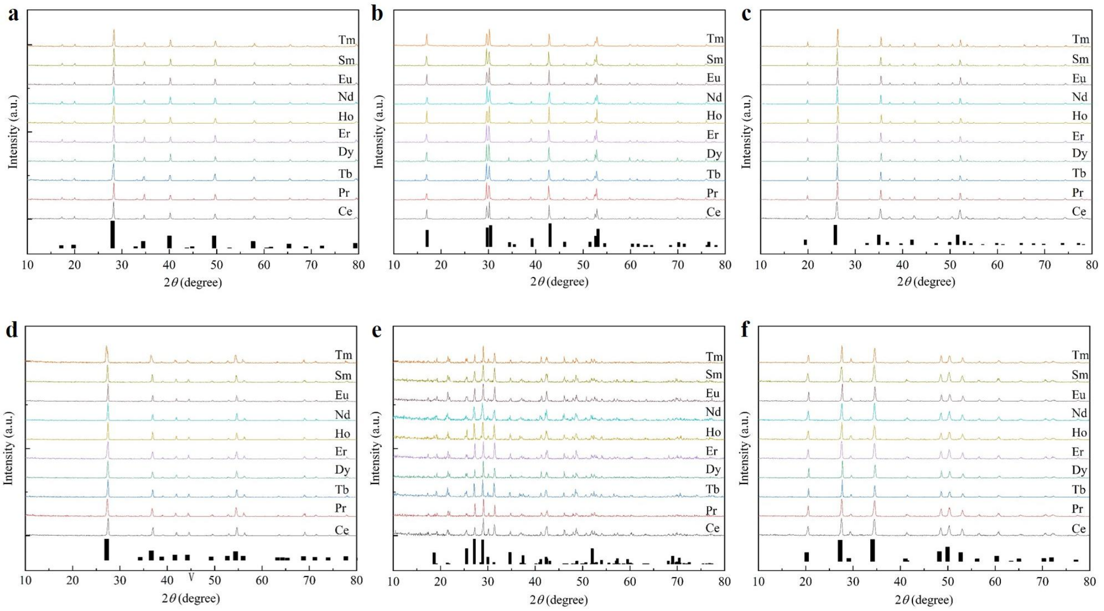

Figure S25. XRD patterns of **(a)** Cs_2NaYF_6 doped with different trivalent lanthanides, the reference standard data is from no. PDF#20-1214; **(b)** $NaYF_4$ doped with different trivalent lanthanides, the reference standard data is from no. PDF#16-0334; **(c)** YPO_4 doped with different trivalent lanthanides, the reference standard data is from no. PDF#11-0254; **(d)** $ScPO_4$ doped with different trivalent lanthanides, the reference standard data is from no. PDF#08-0047; **(e)** $LaPO_4$ doped with different trivalent lanthanides, the reference standard data is from no. PDF#46-1326; **(f)** YBO_3 doped with different trivalent lanthanides, the reference standard data is from no. PDF#16-0277.

1. E. Nakazawa, The lowest 4f-to-5d and charge-transfer transitions of rare earth ions in YPO_4 hosts. *Journal of Luminescence* **100**, 89-96 (2002).
2. T. Watrous-Kelley, A. L. Diaz, T. A. Dang. Quantitative determination of nonradiative host-to-activator energy transfer efficiencies in YBO_3:Eu^{3+} and Y_2O_3:Eu^{3+} under vacuum ultraviolet excitation. *Chemistry of Materials* **18**, 3130-3136 (2006).
